# Maternal and pregnancy factors contributing to the association between area deprivation and infant mortality in England: a retrospective cohort study

**DOI:** 10.1016/j.lanepe.2024.101075

**Published:** 2024-10-01

**Authors:** Frederick K. Ho, Max Allan, Hui Shao, Kenneth K.C. Man, Bhautesh D. Jani, Donald Lyall, Claire Hastie, Michael Fleming, Daniel Mackay, John G.F. Cleland, Christian Delles, Ruth Dundas, Jim Lewsey, Patrick Ip, Ian Wong, Paul Welsh, Anna Pearce, Charlotte M. Wright, Helen Minnis, S Vittal Katikireddi, Jill P. Pell

**Affiliations:** a School of Health and Wellbeing, University of Glasgow, UK; b School of Pharmacy, University College London, UK; c School of Cardiovascular and Metabolic Health, University of Glasgow, UK; d Department of Paediatrics and Adolescent Medicine, Li Ka Shing Faculty of Medicine, The University of Hong Kong, Hong Kong; e Department of Paediatrics and Adolescent Medicine, Hong Kong Children's Hospital, Hong Kong; f Department of Pharmacology and Pharmacy, Li Ka Shing Faculty of Medicine, The University of Hong Kong, Hong Kong; g Aston School of Pharmacy, Aston University, UK; h School of Medicine, Dentistry & Nursing, University of Glasgow, UK

**Keywords:** Health inequality, Socioeconomic deprivation, Infant mortality, Cohort study

## Abstract

**Background:**

Socioeconomic inequality in infant mortality in the UK is rising. This study aims to identify contributory maternal and pregnancy factors that can explain the known association between area deprivation and infant mortality.

**Methods:**

A cohort study was conducted using Clinical Practice Research Datalink (CPRD) primary care data between 2004 and 2019 linked to the Index of Multiple Deprivation (IMD), and infant mortality from the Office for National Statistics death data. Potential maternal and pregnancy contributory factors included: maternal age, prior maternal health conditions, pregnancy lifestyle factors and complications, use of medications during pregnancy, and characteristics of birth. Counterfactual-based decomposition analysis was used to quantify the relative importance of equalising these factors to reduce inequalities in infant mortality.

**Findings:**

A total of 392,606 mother-child dyads were included in this study. The overall risk of infant mortality was greatest for individuals in the most deprived quintile (risk ratio 2.13 [95% CI 1.58–2.90]; risk difference 6.6 [3.8–8.8] per 10,000 live births) compared with the least deprived. Four contributory factors were identified as potentially important: preterm birth (Proportion eliminated [PE] 15.25% [95% CI 9.44–24.12%]), smoking during pregnancy (PE 13.61% [95% CI 3.96–80.97%]), maternal age <20 years at childbirth (PE 10.52% [95% CI 2.93–21.35%]) and maternal depression (PE 9.13% [95% CI 4.47–14.93%]). These collectively accounted for more than one-third of the socioeconomic inequality in mortality.

**Interpretation:**

Multifactorial interventions targeting maternal mental health, smoking, teenage pregnancy and preterm birth may mitigate a proportion of the effects of socioeconomic inequality but targeting these, alone, will not stem the rise in infant mortality. Structural efforts to reduce socioeconomic inequalities will also be required to prevent these excess infant deaths.

**Funding:**

10.13039/501100000265UK Medical Research Council, Scottish Chief Scientist Office, 10.13039/100010269Wellcome Trust.

**Research in context**

**Evidence before this study**

We searched MEDLINE and Google Scholar with the search terms (“inequalit∗” OR “depriv∗” OR “socioeconomic”) AND (“infant mortality” OR “infant death”) AND (“mechanis∗” OR “mediat∗” OR “modifiable”) for related articles from database inception to 31st March 2023, published in English. Current available evidence is either from small scale cohort studies, or large cohort studies that examined a small, arbitrarily selected set of contributory factors. A Danish cohort study has shown that preterm birth is a strong mediator between maternal education level and infant mortality. However, there were no studies that systematically examined the extent to which inequality could be mitigated through a holistic set of contributory factors.

**Added value of this study**

In this large cohort study of 392,606 mother-child dyads in England, we showed a marked inequality in infant mortality risk. Of the 23 factors examined, 4 factors were identified as potentially important: maternal depression, preterm birth, smoking during pregnancy, and maternal age <20 years at childbirth. These four collectively accounted for almost one-third of the socioeconomic inequality in mortality.

**Implications of all the available evidence**

In the absence of upstream interventions on deprivation, multifactorial interventions, targeting maternal mental health and smoking, and teenage pregnancy could mitigate, but not eliminate the effects of socioeconomic inequality.

## Introduction

As of 2021, England and Wales was ranked 29th out of 38 OECD countries in infant mortality rate^1^ with the latest estimate being 3.7 per 1000 live-births.^2^ While there was improvement in infant mortality from the 1980s to early 2010s, this has plateaued since 2014^2^ with an actual increase in areas with high deprivation.^3^ The infant mortality per 1000 live-births in the most deprived decile increased from 5.3 in 2018 to 5.5 in 2021, while the numbers in the least deprived decile dropped from 2.7 in 2018 to 2.5 in 2021.^2^

It is well known that infant mortality is strongly related to socioeconomic position,^4^ but it is also related to many health behaviours and social factors, that themselves have strong socioeconomic gradients. Teenage pregnancy, mental health conditions, obesity, and lifestyles factors, such as smoking and alcohol consumption during pregnancy, are more prevalent among women in poorer areas, and predispose to unfavourable health in their children.^5^^,^^6^ In addition to the access to and the quality of medical care, and maternal health,^4^ maternal characteristics, prenatal exposures, and pregnancy outcomes also impact infant mortality and vary with deprivation levels.^5^

If these lifestyle factors largely explain the differences in mortality, interventions with downstream factors may help reduce inequality, accompanying efforts to target the fundamental causes.^7^ Socioeconomic inequality and material deprivation have not shown meaningful improvement over the past two decades, and have even widened in recent years.^8^ Over the same period many important contributory factors for infant mortality have become generally less prevalent (e.g. smoking,^9^ teenage pregnancy^10^).

An important question, therefore, is how much of the inequality in infant mortality a reflection of socioeconomically patterned health behaviours and how much infant mortality is related to other specific characteristics that may be amenable to targeted intervention. In order to examine this a large, well phenotyped dataset is required, to give sufficient power to identify individual contributory factors. Therefore, the aim of this study was to identify and quantify factors that contribute to socioeconomic inequalities in infant mortality using the linked Clinical Practice Research Datalink (CPRD) data.

## Methods

### Study design

A population cohort study was undertaken using maternal and pregnancy data recorded in the CPRD GOLD for births between 2004 and 2019 linked to area deprivation and death data. The study was ended in 2019 because babies who were conceived during Covid-19 restriction were found to be socioeconomically more advantaged than those from those prior to Covid-19.^11^

**Fig. 1:**
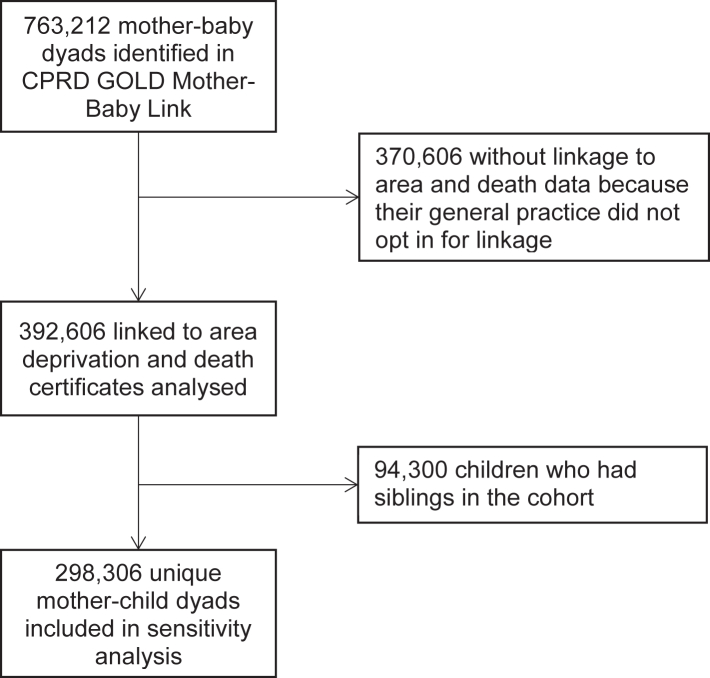
Participant flowchart.

CPRD GOLD is a primary care database of pseudonymised medical records obtained from general practitioners.^12^ As of 2015, approximately 6.9% of the UK population are included in the database.^12^ CPRD with linked data was found to be representative of the UK population in terms of ethnicity and area deprivation.^13^^,^^14^ Within CPRD, full postcode of residence has already been used to derive IMD, obviating the need to share postcodes with researchers which would risk identification of individuals.^12^ In the CPRD data extract for this project, 763,212 mother-baby dyads were identified in the CPRD GOLD Mother-Baby Link, of whom 392,606 were in practices that had opted in for linkage with area deprivation and death data (Fig. 1). Because written consent of participants’ data use in research were obtained during their registration with a general practitioner practice, the CPRD GOLD Mother-Baby Link included only mothers and babies who were both registered. This will have excluded babies who died soon after birth as they would not have been registered with a general practitioner practice.^15^

### Outcome

Office for National Statistics (ONS) death data for England were used to ascertain the outcome, infant mortality, which was defined as any deaths occurring before the age of 1 year. The ONS included all mandatory death records in England and should provide complete data on death. These were categorised into neonatal mortality (death at or before 28 days of age) and postneonatal mortality (deaths at 29 days to before 1 years of age).

### Exposure

Socioeconomic position (SEP) was measured using the Index of Multiple Deprivation (IMD) version 2010, with mother-child dyads assigned an IMD score based on their home address. This measures multiple deprivation at the Lower layer Super Output Area (LSOA). Which includes between 400 and 1200 households with a population between 1000 and 3000.^16^ The IMD is derived from aggregated data for each LSOA across seven domains: income deprivation; employment deprivation; health deprivation and disability; education, skills and training deprivation; barriers to housing and services; crime; and living environment deprivation.^16^ LSOA level data for the construction of IMD was from mid-2008 ONS estimates. IMD scores were categorised into general population quintiles from Q1 (least deprived) to Q5 (most deprived).

### Contributory factors

Birth defects, prematurity-related conditions, and antenatal infections, were the leading causes of infant mortality, accounting for 80% of the cases in England.^2^ Published reviews^17^, ^18^, ^19^ were used to identify the contributory factors known to be associated with these causes. Based on the reviews, a range of factors were derived from primary care records and included in the analyses, selected as they may lie on the causal pathways between SEP and infant mortality. These were birth defects and preterm births,^20^^,^^21^ and their contributory factors: maternal age at childbirth (<20, or ≥40 years), maternal conditions prior to childbirth (anxiety, depression, obesity, heart conditions), pregnancy factors (any record of drinking alcohol or smoking, urinary tract infection [UTI], fever, hyperemesis gravidarum, hypertension, pre-eclampsia, gestational diabetes), any prescription of psychotropic medications during pregnancy (SSRI, other antidepressants, antipsychotic, and benzodiazepine), characteristics of childbirth (preterm birth [gestational age <37 weeks], post-term birth [gestational age ≥42 weeks], and multiple pregnancy), and parity (first live birth). Ascertainment of these factors from primary care records were primarily based on published sources (Supplementary Table S1).

### Data analyses

Descriptive statistics for contributory factors were shown as mean (SD) or number (%) for continuous and categorical variables respectively. These were also reported by IMD quintiles.

The association between IMD quintiles and infant mortality was estimated using a Poisson regression model with Q1 (least deprived) treated as the referent category. This regression model accommodates binary outcome (death vs. alive) by incorporating a robust standard error and produces relative risk estimates.^22^ The outputs were expressed as risk ratio (RR) and 95% confidence interval (CI). In addition, the relative index of inequality (RII) and slope index of inequality (SII) were derived from Poisson models on multiplicative and additive scales^23^ to indicate relative and absolute inequality respectively. RII and SII provide single measures to quantify absolute and relative inequality given the gradient of inequality across IMD quintiles. In the calculation of RII and SII, IMD quintile variable are scaled to a range of 0 (Q1, least deprived) and 1 (Q5, most deprived) so that the regression coefficients represent inequality over the whole spectrum of IMD.

The contributing factors are epidemiological mediators between SEP (proxied by area IMD) and infant mortality. Because there are a large number of them, it is hard to derive a reasonable causal hypothesis. Instead, we used a univariable analysis to identify candidates for multivariable decomposition analysis. This was used to decompose the total association between IMD quintile and infant mortality by natural direct effect and natural indirect effect, assuming causality after adjustment.^24^ The natural indirect effect of a contributory factor indicates the association of IMD quintile on infant mortality operates via that contributory factor. The natural direct effect is the effect of IMD quintile on infant mortality does not operate via that contributory factor. In addition, proportion eliminated (PE) was calculated, being the proportion of the total effect explained by this natural indirect effect.^25^ In the univariable analysis, no confounders were adjusted, and this therefore only serves as a crude estimate. Contributory factors were selected for multivariable analysis if their PE is at least 5%, and with a 95% CI not overlapping with null.

The multivariable analysis was conducted based on the direct acyclic graph (DAG) shown in Supplementary Figure S1, and confounders between contributory factor and infant mortality were adjusted accordingly. Detailed adjustment models for each contributory factor are in the Supplementary Table S2. The causal assumption was based on published observational studies and Mendelian randomisation studies if available, e.g. depression and smoking,^26^ depression and teenage pregnancy.^27^ All decomposition models were estimated using g-formula approach with 2000 bootstrap samples to estimates 95% CIs and p-values. Both outcome and mediator models were based on quasi-Poisson regression.

Three additional analyses were conducted. Firstly, we conducted a period-specific analysis on the prevalence of contributory factors as well as the decomposition analysis for 2004–2011 and 2012–2019 to examine if there were any changes. The cut-off is the mid-point of the study period. Secondly, the decomposition analysis was also repeated for children who survived the neonatal period (28 days) as the study population to examine how the inclusion criteria might have affected the results. Thirdly, this cohort included some multiple children from the same mother, which might have violated the assumption of independent observations in regression analysis. Even though the robust standard errors and bootstrap CIs should not be affected by that, we conducted a sensitivity analysis, including only one random child from each mother. This analysis included 298,306 mother-child dyads (Fig. 1).

All analyses were conducted using R version 4.1.2 with the package CMAverse.

### Role of funding source

The funders have no role in data collection, analysis, interpretation, writing of the manuscript, or the decision to submit.

## Results

### Descriptive statistics

This study included 392,606 live births that occurred between 2004 and 2019. Overall, 343 infants died before 1 year old, of whom 64 died at or before 28 days of age and 279 died from 29 days to before 1 years. The overall infant mortality rate was 8.74, neonatal mortality rate was 1.63, and postneonatal mortality rate of 7.11 per 10,000 live births. Overall, 4.7% of the mothers were younger than 20 years old at childbirth. Depression was the most common condition prior to childbirth (34%). Drinking alcohol and smoking during pregnancy were 8.5% and 7.6% respectively. UTI was the most prevalent pregnancy complication (8.2%), followed by hyperemesis gravidarum (3.2%) and anaemia (2.4%). SSRIs were the most commonly used psychotropic medication during pregnancy (3.5%). Overall, 3.5% of the surviving live births were preterm. Women living in the most deprived quintiles were younger, more likely to have anxiety, depression, and obesity, more likely to smoke during pregnancy, have UTI, hyperemesis gravidarum, anaemia, prescribed SSRIs, and have a preterm birth (Table 1). The prevalence of contributory factors was generally consistent between the two periods (Supplementary Tables S3 and S4), except for: maternal age <20 (5.4% in 2004–2011; 3.5% in 2012–2019), smoking during pregnancy (7.9% in 2004–2011; 6.9% in 2012–2019), and use of SSRI (3.0% in 2004–2011; 4.6% in 2012–2019).

**Table 1:** Participants characteristics.

	Overall	IMD quintiles				
		Q1 least deprived	Q2	Q3	Q4	Q5 most deprived
Total	392,606	84,554 (21.5%)	79,511 (20.3%)	79,511 (20.3%)	81,115 (20.7%)	73,646 (18.8%)
Mean (SD) maternal age at childbirth	29.7 (5.9)	31.8 (5.2)	30.8 (5.5)	29.6 (5.8)	28.5 (5.9)	27.3 (6.0)
<20	18,567 (4.7%)	1339 (1.6%)	2137 (2.7%)	3159 (4.3%)	5181 (6.4%)	6751 (9.2%)
≥40	16,294 (4.2%)	4907 (5.8%)	3932 (4.9%)	2858 (3.9%)	2601 (3.2%)	1996 (2.7%)
First live birth	217,090 (55%)	44,019 (52%)	43,506 (55%)	41,484 (56%)	46,379 (57%)	41,702 (57%)
Prior maternal conditions						
Anxiety	89,273 (23%)	18,432 (22%)	17,837 (22%)	16,952 (23%)	18,455 (23%)	17,597 (24%)
Depression	134,032 (34%)	24,982 (30%)	25,409 (32%)	25,511 (35%)	29,700 (37%)	28,430 (39%)
Obesity	102,715 (26%)	16,545 (20%)	18,105 (23%)	19,194 (26%)	24,556 (30%)	24,315 (33%)
Heart conditions	152 (<0.1%)	38 (<0.1%)	21 (<0.1%)	29 (<0.1%)	30 (<0.1%)	34 (<0.1%)
Pregnancy factors						
Alcohol drinking	33,478 (8.5%)	7393 (8.7%)	7186 (9.0%)	6125 (8.3%)	6938 (8.6%)	5836 (7.9%)
Smoking	29,667 (7.6%)	2679 (3.2%)	3965 (5.0%)	5439 (7.4%)	7992 (9.9%)	9592 (13%)
Urinary tract infection	32,111 (8.2%)	5626 (6.7%)	5923 (7.4%)	5958 (8.1%)	7219 (8.9%)	7385 (10%)
Fever	1197 (0.3%)	253 (0.3%)	208 (0.3%)	242 (0.3%)	242 (0.3%)	252 (0.3%)
Hyperemesis gravidarum	12,578 (3.2%)	2320 (2.7%)	2249 (2.8%)	2380 (3.2%)	2833 (3.5%)	2796 (3.8%)
Anaemia	9235 (2.4%)	1839 (2.2%)	1472 (1.9%)	1752 (2.4%)	2057 (2.5%)	2115 (2.9%)
Hypertension	3875 (1.0%)	724 (0.9%)	727 (0.9%)	731 (1.0%)	891 (1.1%)	802 (1.1%)
Pre-eclampsia	986 (0.3%)	227 (0.3%)	227 (0.3%)	159 (0.2%)	218 (0.3%)	155 (0.2%)
Gestational diabetes	3875 (1.0%)	724 (0.9%)	727 (0.9%)	731 (1.0%)	891 (1.1%)	802 (1.1%)
Use of medications during pregnancy						
SSRI	13,933 (3.5%)	2118 (2.5%)	2438 (3.1%)	2686 (3.6%)	3304 (4.1%)	3387 (4.6%)
Other antidepressants	4007 (1.0%)	612 (0.7%)	644 (0.8%)	754 (1.0%)	982 (1.2%)	1015 (1.4%)
Antipsychotics	9679 (2.5%)	1787 (2.1%)	1671 (2.1%)	1849 (2.5%)	2261 (2.8%)	2111 (2.9%)
Benzodiazepines	2491 (0.6%)	466 (0.6%)	483 (0.6%)	513 (0.7%)	569 (0.7%)	460 (0.6%)
Birth outcome						
Preterm birth	13,903 (3.5%)	2629 (3.1%)	2468 (3.1%)	2560 (3.5%)	3090 (3.8%)	3156 (4.3%)
Postterm birth	35,912 (9.1%)	8542 (10%)	7242 (9.1%)	6865 (9.3%)	7376 (9.1%)	5887 (8.0%)
Multiple pregnancy	3298 (0.8%)	840 (1.0%)	718 (0.9%)	635 (0.9%)	604 (0.7%)	501 (0.7%)
Any birth defects	35,113 (8.9%)	8603 (10%)	7580 (9.5%)	6380 (8.6%)	6688 (8.2%)	5862 (8.0%)

### IMD and infant mortality

The numbers and rates of infant mortality overall and by IMD quintile are shown in Table 2. The RII was 2.13 (95% CI 1.58–2.90) and the SII was 6.30 (95% CI 3.79–8.81) per 10,000 live births. There was also a dose-response relationship between IMD quintile and infant mortality, the higher the deprivation quintile, the higher the infant mortality. Overall and IMD quintile specific infant mortality rate, and SII were slightly lower in 2012–2019 than in 2004–2011, even though RII was slightly increased.

**Table 2:** Infant mortality rates (per 10,000 live births) and overall inequality measures.

	Numbers	Deaths	Infant mortality rate (95% CI)	RII (95% CI)	SII (95% CI)
**Overall**	392,606	343	8.74 (7.85–9.72)	2.13 (1.58–2.90)	6.30 (3.79–8.81)
Q1	84,554	51	6.03 (4.54–8.00)		
Q2	79,511	54	6.79 (5.15–8.93)		
Q3	73,780	63	8.54 (6.62–11.00)		
Q4	81,115	82	10.11 (8.09–12.61)		
Q5	73,646	93	12.63 (10.25–15.54)		
**2004**–**2011**	251,671	244	9.70 (8.53–11.01)	2.10 (1.46–3.01)	6.82 (3.50–10.13)
Q1	54,370	37	6.81 (4.86–9.48)		
Q2	51,214	38	7.42 (5.32–10.29)		
Q3	47,755	49	10.26 (7.67–13.68)		
Q4	51,924	53	10.21 (7.72–13.46)		
Q5	46,408	67	14.44 (11.28–18.45)		
**2012**–**2019**	140,935	99	7.02 (5.74–8.59)	2.26 (1.29–4.02)	5.45 (1.71–9.17)
Q1	30,184	14	4.64 (2.64–7.99)		
Q2	28,297	16	5.65 (3.35–9.40)		
Q3	26,025	14	5.38 (3.06–9.27)		
Q4	29,191	29	9.93 (6.78–14.47)		
Q5	27,238	26	9.55 (6.37–14.20)		

RII: relative index of inequality; SII: slope index of inequality.

### Decomposition via contributory factors

The univariable decomposition analysis results are shown in Table 3. Four contributory factors reached the pre-specified threshold: Maternal age <20 (PE 11.41, 95% CI 4.63, 23.53), depression (proportion eliminated (PE) 8.85, 95% CI 4.37, 16.91), smoking during pregnancy (PE 15.47, 95% CI 6.67, 28.13), and preterm birth (PE 15.75, 95% CI 10.30, 23.81).

**Table 3:** Univariable decomposition analysis of excess infant mortality risk of IMD Q5 compared with Q1.

	Natural direct effect	Natural indirect effect	Total effect	% eliminated
Maternal age at childbirth				
**<20**	**2.01 (1.48, 2.81)**	**1.06 (1.02, 1.13)**	**2.14 (1.57, 2.95)**	**11.41 (4.63, 23.53)**
≥40	2.15 (1.52, 2.90)	0.99 (0.97, 1.01)	2.13 (1.52, 2.88)	−1.88 (−5.55, 1.88)
First live birth	2.19 (1.59, 3.00)	0.98 (0.97, 0.98)	2.14 (1.56, 2.94)	−0.04 (−0.06, −0.03)
Prior maternal conditions				
Anxiety	2.13 (1.61, 2.81)	1.00 (1.00, 1.01)	2.13 (1.62, 2.82)	0.56 (−0.40, 1.71)
**Depression**	**2.05 (1.47, 2.97)**	**1.05 (1.02, 1.09)**	**2.15 (1.55, 3.10)**	**8.85 (4.37, 16.91)**
Obesity	2.12 (1.53, 2.76)	1.01 (0.98, 1.06)	2.13 (1.54, 2.80)	1.47 (−4.59, 9.81)
Heart conditions	2.13 (1.64, 2.88)	1.00 (1.00, 1.00)	2.13 (1.64, 2.88)	−0.02 (−0.05, 0.03)
Pregnancy factors				
Alcohol drinking	2.13 (1.61, 2.97)	1.00 (1.00, 1.00)	2.13 (1.61, 2.97)	0.07 (−0.59, 0.68)
**Smoking**	**1.97 (1.50, 2.61)**	**1.09 (1.04, 1.15)**	**2.15 (1.62, 2.86)**	**15.47 (6.67, 28.13)**
Urinary tract infection	2.13 (1.61, 3.11)	1.00 (0.99, 1.01)	2.13 (1.61, 3.09)	0.44 (−2.18, 2.83)
Fever	2.13 (1.58, 2.93)	1.00 (1.00, 1.00)	2.13 (1.58, 2.93)	−0.01 (−0.17, 0.26)
Hyperemesis gravidarum	2.13 (1.57, 2.87)	1.00 (0.99, 1.01)	2.13 (1.57, 2.87)	−0.07 (−1.19, 1.25)
Anaemia	2.14 (1.58, 2.75)	1.00 (0.99, 1.00)	2.13 (1.58, 2.75)	−0.25 (−1.26, 0.68)
Hypertension	2.13 (1.57, 2.89)	1.00 (1.00, 1.00)	2.13 (1.57, 2.90)	0.25 (−0.34, 0.91)
Pre-eclampsia	2.14 (1.65, 2.94)	1.00 (0.99, 1.00)	2.13 (1.65, 2.94)	−0.40 (−1.26, 0.00)
Gestational diabetes	2.13 (1.59, 2.82)	1.00 (1.00, 1.01)	2.13 (1.59, 2.81)	0.24 (−0.35, 1.08)
Prescription of psychotropic medications during pregnancy				
SSRI	2.10 (1.55, 2.73)	1.02 (1.00, 1.03)	2.13 (1.58, 2.79)	3.26 (−0.65, 7.97)
Other antidepressants	2.13 (1.56, 2.85)	1.00 (1.00, 1.01)	2.13 (1.57, 2.87)	0.44 (−0.95, 2.36)
Antipsychotics	2.13 (1.56, 2.88)	1.00 (1.00, 1.01)	2.13 (1.56, 2.88)	0.45 (−0.66, 2.13)
Benzodiazepines	2.13 (1.62, 3.02)	1.00 (1.00, 1.00)	2.13 (1.62, 3.03)	0.05 (−0.21, 0.40)
Birth outcome				
**Preterm birth**	**1.99 (1.42, 2.68)**	**1.09 (1.06, 1.13)**	**2.17 (1.54, 3.00)**	**15.75 (10.30, 23.81)**
Postterm birth	2.11 (1.60, 2.97)	1.01 (1.01, 1.01)	2.13 (1.62, 2.99)	1.68 (0.82, 3.05)
Multiple pregnancy	2.14 (1.53, 3.04)	1.00 (0.99, 1.00)	2.13 (1.53, 3.04)	−0.80 (−2.63, −0.03)
Any birth defects	2.25 (1.69, 3.17)	0.93 (0.91, 0.95)	2.10 (1.56, 2.93)	−13.92 (−22.55, −9.13)

Bold font indicates contributory factors with % eliminated ≥5% with 95% CI not overlapping with null.

**Fig. 2:**
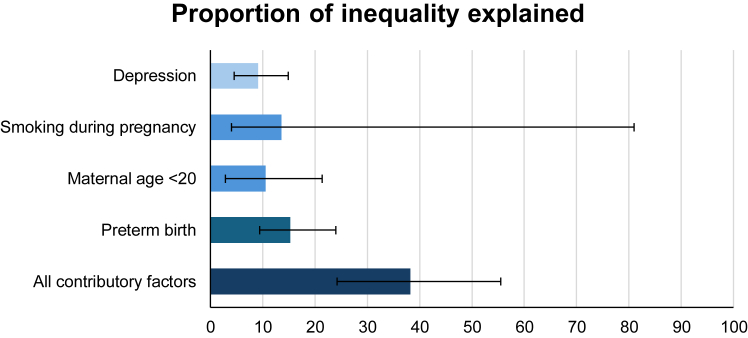
Proportion eliminated in multivariable decomposition analysis. Proportion eliminated indicates the proportion of excess infant mortality risk in IMD Q5 that could be eliminated if the contributory factors were equalised, assuming causality. All contributory factors account for associations between contributory factors so its % eliminated is less than the sum of all five individual factors.

The multivariable decomposition model produced an effect size estimate (RR 2.17, 95% CI 1.65, 2.95) similar to the RII. After adjusting for mediator-outcome confounding, the included contributory factors collectively explained 38.22% of the socioeconomic inequality in infant mortality (Fig. 2) with the following contributions: preterm birth (15.25, 95% CI 9.44, 24.12), smoking during pregnancy (13.61, 95% CI 3.96, 80.97), maternal age <20 (10.52, 95% CI 2.93, 21.35), and maternal depression (9.13, 95% CI 4.47, 1). Two of these (maternal age <20 years at childbirth and smoking during pregnancy) could be deemed to reflect health behaviours and they collectively explained 21.97% of the inequality. Detailed decomposed effects are shown in Supplementary Table S5.

The univariable decomposition analysis showed similar results between the two period (Supplementary Tables S6 and S7). Including only children who survived the neonatal period provided similar findings. Including only unique mother-child dyads, univariable and multivariable decomposition analyses showed similar relative importance in contributory factors but with reduced PEs (Supplementary Tables S9 and S10).

## Discussion

### Principal findings

Over the study period, infants in the most deprived areas had double the mortality rate compared with the least deprived areas with a dose-relationship between deprivation and mortality. Four factors (preterm birth, smoking during pregnancy, low maternal age and maternal depression) were identified that with adjustment each attenuated that association by between 10 and 15%. Two partially behavioural factors, lower maternal age at birth and smoking during pregnancy, collectively explained 22% of the inequality. These findings suggest that interventions targeting behavioural and clinical contributory factors could only mitigate a proportion of the effects of sociodemographic inequality. Structural changes targeting socioeconomic inequality, e.g. increasing investment in people and areas facing adversity, will be required to have a substantial impact on inequality in infant mortality.

### Comparison with existing literature

Our finding of an overall association between deprivation and infant mortality is consistent with many previous studies which have reported associations for both individuals living in areas with a greater deprivation score, and in individuals residing in an area of poverty.^28^ Consistent with our findings, a systematic review on preterm births also suggested that maternal smoking during pregnancy could explain a part of the socioeconomic inequality, but that a large residual inequality existed.^29^ Previous studies^30^ have also suggested that interventions to reduce maternal depression could potentially reduce overall and the socioeconomic patterning of infant mortality.

A study conducted in Denmark suggested that maternal age and preterm birth could explain almost half of the association between low maternal education and infant mortality^31^; this contrasts with our findings that maternal age at childbirth and preterm birth could only explain at most 15% of the excess risk. The discrepancy could be due to the measure of SEP being used (education vs. area deprivation) as well as the lower economic inequality in Denmark.

### Strengths and limitations

While this study provided a comprehensive, robust exploration of maternal and pregnancy factors to explain infant mortality inequality, this study has several limitations. This study cannot establish causality, despite our best effort in addressing confounding between contributory factor and infant mortality. Residual confounding can over- or under-estimate the proportion eliminated depending on the confounder.^32^ However, using routinely collected data reduces the selection and recall biases commonly observed in cohort studies. Patient-level linkage of CPRD and ONS is considered to be the gold standard for measuring mortality rate,^33^ and the lack of linkage for some CPRD patients was because general practices did not opt in for such linkage rather than other systematic reasons. Even though only 6.9% of the UK population are covered by CPRD, their selection is due to the primary care computing system used by general practices, rather than from other systematic factors, and therefore unlikely to introduce bias.^12^

The IMD does not measure participants’ individual-level SEP, but the combination of multiple measures at the small area level provides a measure of SEP that is usually not well captured in routine data. It has been shown in many studies to effectively discriminate between lower and higher individual SEP.^16^ However, it should be cautioned that IMD is an area-level measure and therefore results extrapolating to individual SEP could be subject to ecological fallacy. Ideally, future studies should corroborate our findings using individual-level measures that capture different dimensions of SEP, such as maternal education level or household income, as well as other axes of inequality, such as ethnicity.

Whilst maternal age and prescription will be complete and accurate, some factors may be systematically incomplete or inaccurate, particularly lifestyle factor such as smoking and alcohol, so the total influence of these may well have been underestimated. Notably the smoking prevalence reported in this study was lower than some reports.^34^ Obesity may in other circumstances be under-recorded or rely on self-reported weights, but all pregnant women are routinely weighed and measured, and the prevalence of obesity found are comparable to those reported for pregnant women.^35^ The use of binary contributory variable (e.g. preterm) and a lifelong lookback period for prior conditions (e.g. depression) could result in measurement bias and skewed the estimated importance. In addition, the prevalence of preterm births in this study is lower than in other reports, which could be related to the underreporting or the inclusion criteria described below.

Depression, which was an important contributory factor was recorded as present if the diagnosis had been made at any point up to childbirth and the timing was not taken into account. This makes it more likely that any association has been captured, but where depression may have long preceded pregnancy, this may also include non-causal associations, where depression is in effect a marker for other unmeasured factors.

While CPRD with linked data was found to be representative of the UK population,^13^^,^^14^ the study could not include all infant deaths because babies had to be registered with a general practitioner to be included, and those babies who died soon after birth would not have been registered. As around 75% of all infant mortality occurs in the neonatal period, this resulted in an neonatal mortality rate estimated in this study to be much lower than the UK figure in 2019 (1.6 vs. 29 per 10,000) but a postneonatal mortality rate that was closer (7.1 vs. 10 per 10,000).^2^ Because a higher proportion of neonatal deaths are related immaturity conditions,^2^ some of our results on the relative importance of prematurity could be underestimated in neonatal deaths. The exclusion of some neonatal deaths, many of whom would have been born preterm, is also a reason why the prevalence of preterm birth in this study is lower than in other reports. Indeed, the sensitivity analysis focusing on postneonatal deaths showed a lower PE for preterm births than in the analysis on all infant deaths. But our findings should still be broadly relevant to all infant deaths, as both neonatal and postneonatal deaths share the same two leading causes of deaths (i.e. immaturity related conditions and congenital anomalies), accounting for about 75% of all infant deaths.^2^ In addition, this study might not be able to include a small (but vulnerable) proportion of the population who may not be registered with a GP, e.g. those who are homeless or from traveller communities. Of note, even though the mother-baby linkage was based on a published algorithm which was shown to have high sensitivity and completeness,^36^ it is possible that there existed unlinked babies and those might be particularly vulnerable.

### Implications

Socioeconomic inequalities in health are fundamentally unjust.^37^ As our findings highlight, their association with both maternal health and infant mortality, perpetuate these inequalities between generations. Even considering 24 individual potential contributory factors, only 38% of inequality in infant mortality could be explained. This suggests that higher level structural changes may be of greater importance for levelling up such inequality. These could include increased funding for early year's learning, education, and more high quality and well paid job opportunities in deprived areas, which would both increase the overall health of the population and reduce health inequalities.^38^ Importantly, inequality in the living and working conditions affecting infant health (e.g. antenatal care, health services) and their parents (e.g. parental leave) should be addressed.^39^ The UK government austerity programme officially started in 2010 and impacted social determents of child health as well as child health outcomes.^34^^,^^40^ In addition, institutional racism and other forms of discrimination should be targeted since ethnicity (strongly linked to IMD^41^) are additional sources of inequality in infant mortality.^42^

Given the current persistence of socioeconomic inequalities, our study investigated whether any contributory factors could be identified that might offer other targets for intervention that might reduce the transmission of inequalities to the next generation. Maternal depression prior to childbirth was found to be one of the strongest contributory factors in our study, explaining almost 10% of the excess risk in infant mortality between the most and least deprived fifth of areas. Screening for depression in early pregnancy has been recently shown to be cost-effective^43^ and may be a feasible way to reduce infant mortality. Both psychological and pharmacological treatments for depression are effective^44^^,^^45^ and recent data did not support the causal link between antidepressants use during pregnancy and offspring neurodevelopmental conditions.^46^^,^^47^ However, it should be noted that there is some evidence that both psychological and pharmacological treatments are less effective in people with lower SEP,^48^^,^^49^ and future interventions should ensure they are accessible and acceptable across SEPs. We found no evidence that prenatal psychotropic medication contributed significantly to the observed inequality, though this finding should be corroborated in other studies. On the other hand, depression may be a marker for other unmeasured, or under-reported adversities or risks, such as substance abuse,^50^ poor housing or domestic violence,^51^ in which case even effective treatment may not impact on infant mortality.

The UK still has the highest teenage pregnancy rate in Western Europe^52^ and these result further illustrate how strongly this is socioeconomically patterned.^53^ Pregnancy in adolescence is associated with higher risk of very preterm childbirth (<32 weeks)^53^ which itself strongly increases the risk of infant mortality.^54^ Between 1998 and 2016 under-18 conception rates decreased by 51% in the UK overall, but most markedly in the most deprived areas,^10^ which could partially explain the overall drop in infant mortality over the study period. Teenage conception rates could potentially be decreased further by a combination of enhanced education and contraceptive service provision.^55^ It is important to note that the increased risk is likely due to the underlying circumstances indicated by teenage pregnancy, rather than biological age of the mother.

Smoking during pregnancy was also a powerful contributory factor to inequality in infant mortality. The population smoking prevalence has continued to decline, from 20.2% in 2011 to 12.9% in 2022,^9^ but at the same time inequality has increased. Most recent data in England showed that one-third of smoking adults lived in the most deprived quintile in 2021, up from less than 30% in 2017.^56^ This illustrates the challenge of tackling downstream contributory factors to reduce inequality, but supports the argument for the smoke free generation in a recent UK policy paper.^57^

Interestingly just one contributory factor, birth defects, was slightly *less* common in the poorest quintiles and adjusting for it increased the inequality in mortality by some 12%. This is a puzzling finding. This may be related to the exclusion of severe birth defects resulting in death soon after birth, and areas with lower deprivation having more mothers at advanced age, a risk factor for birth defect.^18^

There are potentially other factors in the medical system that we could not capture in this study which could manifest via multiple other mechanisms.^58^ One example is the use and delivery of antenatal care, which is socioeconomically patterned^59^ and could contribute to better birth and child outcomes, through intervention on maternal depression and smoking, as well as reduce the risk of preterm birth.^60^

### Conclusion

There was marked inequality in infant mortality risk. Efforts to modify downstream factors, multifactorial interventions, targeting maternal mental health and smoking, and teenage pregnancy could potentially mitigate a proportion of inequality, but even if all the identified contributory factors could be fully addressed, a large majority of the inequality would still be present. It is important to continue to focus on and address the fundamental causes of inequality.

## Contributors

FKH and JPP conceptualised the study. FKH analysed the data and wrote the first draft of the manuscript. MA and HS analysed, verified, and interpreted the data, and wrote the first draft the manuscript. All other authors interpreted the data and critically revised the manuscript. FKH is responsible for the decision to submit the manuscript.

## Data sharing statement

Because of the data sharing agreement with CPRD, we are not able to share the data. However, the data can be requested from CPRD directly (https://www.cprd.com/).

## Declaration of interests

FH received research funding from Glasgow Children Hospital Charity, Biotechnology and Biological Sciences Research Council, Understanding Children's Trust, and The Swedish Child Neuropsychiatry Science Foundation, consulting fee from the Chinese University of Hong Kong, and personal honorarium from Sage Publications. KM received research funding from CW Maplethorpe Fellowship, European Commission Horizon 2020, National Institute for Health and Care Research UK, Hong Kong Research Grant Council. JC received research funding from Bristol Myers Squibb, CSL-Vifor, British Heart Foundation, and Pharmacosmos, consulting fee from Pharmacosmos, CSL-Vifor, and Biopeutics, personal honorarium from Pharmacosmos and ABBOTT, travel support from Pharmacosmos, participated on advisory boards of Medtronic, ADAPT-CRT, CMR-Guide, PROTECT-HF, and have stocks or stock options in HeartFelt (non-invasive monitoring), Viscardia (synchronous diaphragmatic pacing). CD received research funding from Wellcome Trust. PI received research funding from Hong Kong Health and Medical Research Fund and Hong Kong General Research Fund. ICKW received research grants from Amgen, Janssen, GSK, Novartis, Pfizer, Bayer and Bristol-Myers Squibb and Takeda, Institute for Health Research in England, European Commission, National Health and Medical Research Council in Australia, The European Union's Seventh Framework Programme for research, technological development, Research Grants Council Hong Kong and Health and Medical Research Fund Hong Kong; consulting fees from IQVIA and World Health Organization; payment for expert testimony for Appeal Court in Hong Kong; serves on advisory committees for Member of Pharmacy and Poisons Board; is a member of the Expert Committee on Clinical Events Assessment Following COVID-19 Immunization; is a member of the Advisory Panel on COVID-19 Vaccines of the Hong Kong Government; is the non-executive director of Jacobson Medical in Hong Kong; and is the founder and director of Therakind Limited (UK), Advance Data Analytics for Medical Science (ADAMS) Limited (HK), Asia Medicine Regulatory Affairs (AMERA) Services Limited and OCUS Innovation Limited (HK, Ireland and UK). PW received research funding from Boehringer Ingelheim, Astrazeneca, Roche diagnostics, Novartis, personal honorarium from Novo Nordisk and Raisio. AP received research funding from Wellcome Trust, MRC, CSO, NIHR, Obesity Action Scotland, Health Foundation, and UKPRP.
