# Supplementary material for: Maternal and pregnancy factors contributing to the association between area deprivation and infant mortality in England: a retrospective cohort study
Source: Lancet Reg Health Eur. 2024 Oct 1;47:101075. doi: 10.1016/j.lanepe.2024.101075 (PMC11670682; doi:10.1016/j.lanepe.2024.101075)
Supplement: Supplementary Figure and Tables [file mmc1.docx]

Supplementary Materials

# Supplementary Figure 1. Causal assumption of the analysis


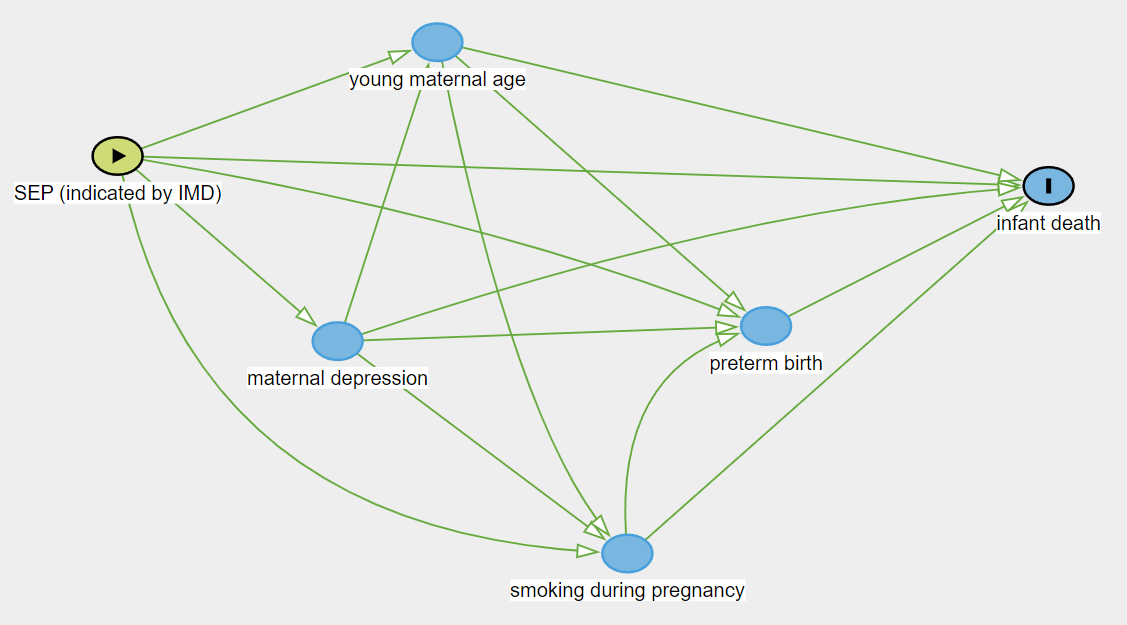


# Supplementary Table 1. Ascertainment of contributory factors

| **Contributory factor** | **Methods of ascertainment** |
| --- | --- |
| Maternal age at childbirth, gestational age, parity, and multiple pregnancy | CPRD Mother-Baby Link |
| Prior maternal conditions | Any relevant primary care records of the mother that occurred before childbirth |
| Anxiety | Read codes v2 ^60^ |
| Depression | Read codes v2 ^60^ |
| Obesity | CPRD med codes, including recorded BMI >= 30 ^61^ |
| Heart conditions | CPRD med codes ^61^ |
| Pregnancy factors | Any relevant primary care records of the mother that occurred during pregnancy |
| Alcohol drinking | Read codes v2 ^62^ |
| Smoking | Read codes v2 ^63^ |
| Urinary tract infection | Read codes v2 ^64^ |
| Fever | Bespoke Read codes v2 |
| Hyperemesis gravidarum | Bespoke Read codes v2 |
| Anaemia | CPRD med codes ^61^ |
| Hypertension | CPRD med codes ^61^ and product codes ^65^ |
| Pre-eclampsia | Bespoke Read codes v2 |
| Gestational diabetes | CPRD med codes ^61^ and product codes ^65^ |
| Prescriptions during pregnancy | Any relevant primary care records of the mother that occurred during pregnancy |
| SSRI | CPRD product codes ^60^ |
| Other antidepressants | CPRD product codes ^60^ |
| Antipsychotics | CPRD product codes ^60^ |
| Benzodiazepines | CPRD product codes ^60^ |
| Any birth defects | Read codes v2 ^66^ |

# Supplementary Table 2. Adjustment models in the association between contributory factors and infant mortality in main analysis

| **Contributory factor** | **Adjusted factors** |
| --- | --- |
| Maternal depression | None |
| Young maternal age | Maternal depression |
| Maternal obesity | Maternal depression |
| Smoking during pregnancy | Maternal depression, Young maternal age |
| Preterm birth | Maternal depression, Maternal obesity, Young maternal age, Smoking during pregnancy |

# Supplementary Table 3: Prevalence of contributory factors in 2004-2011

|  | **Overall** | **IMD quintiles** | | | | |
| --- | --- | --- | --- | --- | --- | --- |
|  |  | **Q1 Least deprived** | **Q2** | **Q3** | **Q4** | **Q5 Most deprived** |
| Maternal age at childbirth |  |  |  |  |  |  |
| <20 | 13,645 (5.4%) | 965 (1.8%) | 1,552 (3.0%) | 2,325 (4.9%) | 3,824 (7.4%) | 4,979 (11%) |
| ≥40 | 9,923 (3.9%) | 2,895 (5.3%) | 2,466 (4.8%) | 1,818 (3.8%) | 1,581 (3.0%) | 1,163 (2.5%) |
| First live birth | 138,262 (55%) | 28,063 (52%) | 27,969 (55%) | 26,547 (56%) | 29,572 (57%) | 26,111 (56%) |
| Prior maternal conditions |  |  |  |  |  |  |
| Anxiety | 56,650 (23%) | 11,652 (21%) | 11,274 (22%) | 10,725 (22%) | 11,760 (23%) | 11,239 (24%) |
| Depression | 88,671 (35%) | 16,480 (30%) | 16,822 (33%) | 16,944 (35%) | 19,647 (38%) | 18,778 (40%) |
| Obesity | 66,706 (27%) | 10,836 (20%) | 11,893 (23%) | 12,479 (26%) | 15,891 (31%) | 15,607 (34%) |
| Heart conditions | 119 (<0.1%) | 24 (<0.1%) | 17 (<0.1%) | 25 (<0.1%) | 24 (<0.1%) | 29 (<0.1%) |
| Pregnancy factors |  |  |  |  |  |  |
| Alcohol drinking | 21,813 (8.7%) | 4,851 (8.9%) | 4,679 (9.1%) | 4,029 (8.4%) | 4,460 (8.6%) | 3,794 (8.2%) |
| Smoking | 19,981 (7.9%) | 1,807 (3.3%) | 2,697 (5.3%) | 3,724 (7.8%) | 5,331 (10%) | 6,422 (14%) |
| Urinary tract infection | 20,156 (8.0%) | 3,506 (6.4%) | 3,769 (7.4%) | 3,790 (7.9%) | 4,566 (8.8%) | 4,525 (9.8%) |
| Fever | 776 (0.3%) | 153 (0.3%) | 137 (0.3%) | 159 (0.3%) | 149 (0.3%) | 178 (0.4%) |
| Hyperemesis gravidarum | 7,387 (2.9%) | 1,368 (2.5%) | 1,350 (2.6%) | 1,415 (3.0%) | 1,668 (3.2%) | 1,586 (3.4%) |
| Anaemia | 6,489 (2.6%) | 1,334 (2.5%) | 1,035 (2.0%) | 1,233 (2.6%) | 1,436 (2.8%) | 1,451 (3.1%) |
| Hypertension | 2,297 (0.9%) | 446 (0.8%) | 451 (0.9%) | 426 (0.9%) | 507 (1.0%) | 467 (1.0%) |
| Pre-eclampsia | 642 (0.3%) | 142 (0.3%) | 154 (0.3%) | 96 (0.2%) | 150 (0.3%) | 100 (0.2%) |
| Gestational diabetes | 2,297 (0.9%) | 446 (0.8%) | 451 (0.9%) | 426 (0.9%) | 507 (1.0%) | 467 (1.0%) |
| Use of medications during pregnancy |  |  |  |  |  |  |
| SSRI | 7,511 (3.0%) | 1,133 (2.1%) | 1,285 (2.5%) | 1,456 (3.0%) | 1,786 (3.4%) | 1,851 (4.0%) |
| Other antidepressants | 2,273 (0.9%) | 368 (0.7%) | 386 (0.8%) | 397 (0.8%) | 573 (1.1%) | 549 (1.2%) |
| Antipsychotics | 6,147 (2.4%) | 1,142 (2.1%) | 1,065 (2.1%) | 1,189 (2.5%) | 1,440 (2.8%) | 1,311 (2.8%) |
| Benzodiazepines | 1,683 (0.7%) | 325 (0.6%) | 325 (0.6%) | 346 (0.7%) | 388 (0.7%) | 299 (0.6%) |
| Birth outcome |  |  |  |  |  |  |
| Preterm birth | 8,762 (3.5%) | 1,720 (3.2%) | 1,558 (3.0%) | 1,617 (3.4%) | 1,920 (3.7%) | 1,947 (4.2%) |
| Postterm birth | 23,416 (9.3%) | 5,667 (10%) | 4,673 (9.1%) | 4,513 (9.5%) | 4,798 (9.2%) | 3,765 (8.1%) |
| Multiple pregnancy | 2,139 (0.8%) | 543 (1.0%) | 478 (0.9%) | 435 (0.9%) | 377 (0.7%) | 306 (0.7%) |
| Any birth defects | 22,553 (9.0%) | 5,441 (10%) | 4,877 (9.5%) | 4,192 (8.8%) | 4,330 (8.3%) | 3,713 (8.0%) |

# Supplementary Table 4: Prevalence of contributory factors in 2012-2019

|  | **Overall** | **IMD quintiles** | | | | |
| --- | --- | --- | --- | --- | --- | --- |
|  |  | **Q1 Least deprived** | **Q2** | **Q3** | **Q4** | **Q5 Most deprived** |
| Maternal age at childbirth |  |  |  |  |  |  |
| <20 | 4,922 (3.5%) | 374 (1.2%) | 585 (2.1%) | 834 (3.2%) | 1,357 (4.6%) | 1,772 (6.5%) |
| ≥40 | 6,371 (4.5%) | 2,012 (6.7%) | 1,466 (5.2%) | 1,040 (4.0%) | 1,020 (3.5%) | 833 (3.1%) |
| First live birth | 78,828 (56%) | 15,956 (53%) | 15,537 (55%) | 14,937 (57%) | 16,807 (58%) | 15,591 (57%) |
| Prior maternal conditions |  |  |  |  |  |  |
| Anxiety | 32,623 (23%) | 6,780 (22%) | 6,563 (23%) | 6,227 (24%) | 6,695 (23%) | 6,358 (23%) |
| Depression | 45,361 (32%) | 8,502 (28%) | 8,587 (30%) | 8,567 (33%) | 10,053 (34%) | 9,652 (35%) |
| Obesity | 36,009 (26%) | 5,709 (19%) | 6,212 (22%) | 6,715 (26%) | 8,665 (30%) | 8,708 (32%) |
| Heart conditions | 33 (<0.1%) | 14 (<0.1%) | <10 | <10 | <10 | <10 |
| Pregnancy factors |  |  |  |  |  |  |
| Alcohol drinking | 11,665 (8.3%) | 2,542 (8.4%) | 2,507 (8.9%) | 2,096 (8.1%) | 2,478 (8.5%) | 2,042 (7.5%) |
| Smoking | 9,686 (6.9%) | 872 (2.9%) | 1,268 (4.5%) | 1,715 (6.6%) | 2,661 (9.1%) | 3,170 (12%) |
| Urinary tract infection | 11,955 (8.5%) | 2,120 (7.0%) | 2,154 (7.6%) | 2,168 (8.3%) | 2,653 (9.1%) | 2,860 (11%) |
| Fever | 421 (0.3%) | 100 (0.3%) | 71 (0.3%) | 83 (0.3%) | 93 (0.3%) | 74 (0.3%) |
| Hyperemesis gravidarum | 5,191 (3.7%) | 952 (3.2%) | 899 (3.2%) | 965 (3.7%) | 1,165 (4.0%) | 1,210 (4.4%) |
| Anaemia | 2,746 (1.9%) | 505 (1.7%) | 437 (1.5%) | 519 (2.0%) | 621 (2.1%) | 664 (2.4%) |
| Hypertension | 1,578 (1.1%) | 278 (0.9%) | 276 (1.0%) | 305 (1.2%) | 384 (1.3%) | 335 (1.2%) |
| Pre-eclampsia | 344 (0.2%) | 85 (0.3%) | 73 (0.3%) | 63 (0.2%) | 68 (0.2%) | 55 (0.2%) |
| Gestational diabetes | 1,578 (1.1%) | 278 (0.9%) | 276 (1.0%) | 305 (1.2%) | 384 (1.3%) | 335 (1.2%) |
| Use of medications during pregnancy |  |  |  |  |  |  |
| SSRI | 6,422 (4.6%) | 985 (3.3%) | 1,153 (4.1%) | 1,230 (4.7%) | 1,518 (5.2%) | 1,536 (5.6%) |
| Other antidepressants | 1,734 (1.2%) | 244 (0.8%) | 258 (0.9%) | 357 (1.4%) | 409 (1.4%) | 466 (1.7%) |
| Antipsychotics | 3,532 (2.5%) | 645 (2.1%) | 606 (2.1%) | 660 (2.5%) | 821 (2.8%) | 800 (2.9%) |
| Benzodiazepines | 808 (0.6%) | 141 (0.5%) | 158 (0.6%) | 167 (0.6%) | 181 (0.6%) | 161 (0.6%) |
| Birth outcome |  |  |  |  |  |  |
| Preterm birth | 5,141 (3.6%) | 909 (3.0%) | 910 (3.2%) | 943 (3.6%) | 1,170 (4.0%) | 1,209 (4.4%) |
| Postterm birth | 12,496 (8.9%) | 2,875 (9.5%) | 2,569 (9.1%) | 2,352 (9.0%) | 2,578 (8.8%) | 2,122 (7.8%) |
| Multiple pregnancy | 1,159 (0.8%) | 297 (1.0%) | 240 (0.8%) | 200 (0.8%) | 227 (0.8%) | 195 (0.7%) |
| Any birth defects | 12,560 (8.9%) | 3,162 (10%) | 2,703 (9.6%) | 2,188 (8.4%) | 2,358 (8.1%) | 2,149 (7.9%) |

# Supplementary Table 5. All estimates from the multivariable decomposition analysis of excess infant mortality risk of IMD Q5 compared with Q1

|  | **Natural direct effect** | **Natural indirect effect** | **Total effect** | **% eliminated** |
| --- | --- | --- | --- | --- |
| All contributory factors | 1.73 (1.32, 2.46) | 1.26 (1.17, 1.34) | 2.17 (1.65, 2.95) | 38.22 (24.20, 55.45) |
| Depression | 2.05 (1.53, 2.82) | 1.05 (1.02, 1.09) | 2.15 (1.61, 2.95) | 9.13 (4.47, 14.93) |
| Behaviour-related factors | 1.91 (1.42, 2.73) | 1.13 (1.06, 1.29) | 2.16 (1.67, 3.25) | 21.94 (10.51, 45.87) |
| Maternal age < 20 | 2.03 (1.51, 2.76) | 1.06 (1.02, 1.11) | 2.16 (1.61, 2.93) | 10.52 (2.93, 21.35) |
| Smoking during pregnancy | 2.01 (1.54, 2.73) | 1.08 (1.02, 2.91) | 2.17 (1.64, 5.28) | 13.61 (3.96, 80.97) |
| Preterm birth | 2.00 (1.41, 2.82) | 1.09 (1.06, 1.13) | 2.18 (1.54, 3.09) | 15.25 (9.44, 24.12) |

# Supplementary Table 6. Univariable decomposition analysis of excess infant mortality risk of IMD Q5 compared with Q1 for 2004-2011

|  | Natural direct effect | Natural indirect effect | Total effect | % eliminated |
| --- | --- | --- | --- | --- |
| Maternal age at childbirth |  |  |  |  |
| **<20** | **1.70 (1.21, 2.58)** | **1.09 (1.03, 1.19)** | **1.86 (1.36, 2.82)** | **18.73 (5.63, 43.15)** |
| ≥40 | 1.87 (1.32, 2.72) | 0.99 (0.96, 1.01) | 1.85 (1.30, 2.68) | -2.74 (-8.96, 2.12) |
| First live birth | 1.90 (1.40, 2.51) | 0.98 (0.97, 0.99) | 1.86 (1.37, 2.45) | -4.49 (-8.42, -2.72) |
| Prior maternal conditions |  |  |  |  |
| Anxiety | 1.84 (1.40, 2.58) | 1.00 (1.00, 1.01) | 1.85 (1.41, 2.56) | 0.58 (-0.86, 3.00) |
| **Depression** | **1.79 (1.23, 2.51)** | **1.04 (1.01, 1.08)** | **1.86 (1.28, 2.63)** | **8.09 (2.48, 20.88)** |
| Obesity | 1.83 (1.30, 2.62) | 1.01 (0.97, 1.06) | 1.85 (1.28, 2.64) | 2.51 (-7.95, 16.27) |
| Heart conditions | 1.85 (1.32, 2.60) | 1.00 (1.00, 1.00) | 1.85 (1.32, 2.60) | 0.00 (-0.08, 0.04) |
| Pregnancy factors |  |  |  |  |
| Alcohol drinking | 1.85 (1.36, 2.58) | 1.00 (1.00, 1.00) | 1.85 (1.36, 2.58) | -0.19 (-1.05, 0.68) |
| **Smoking** | **1.73 (1.20, 2.49)** | **1.07 (1.01, 1.15)** | **1.85 (1.29, 2.66)** | **14.44 (1.53, 37.82)** |
| Urinary tract infection | 1.84 (1.35, 2.63) | 1.00 (0.99, 1.02) | 1.85 (1.35, 2.65) | 0.67 (-2.93, 4.41) |
| Fever | 1.85 (1.39, 2.58) | 1.00 (1.00, 1.00) | 1.85 (1.39, 2.57) | -0.21 (-0.40, -0.04) |
| Hyperemesis gravidarum | 1.85 (1.40, 2.67) | 1.00 (0.99, 1.01) | 1.85 (1.40, 2.68) | -0.27 (-2.05, 1.52) |
| Anaemia | 1.86 (1.37, 2.49) | 1.00 (0.99, 1.00) | 1.85 (1.36, 2.48) | -0.78 (-1.82, 0.07) |
| Hypertension | 1.85 (1.33, 2.78) | 1.00 (1.00, 1.00) | 1.85 (1.33, 2.77) | -0.09 (-0.72, 0.68) |
| Pre-eclampsia | 1.85 (1.29, 2.84) | 1.00 (0.99, 1.00) | 1.85 (1.29, 2.83) | -0.40 (-1.92, 0.10) |
| Gestational diabetes | 1.85 (1.43, 2.60) | 1.00 (1.00, 1.00) | 1.85 (1.43, 2.60) | -0.07 (-0.84, 0.64) |
| Prescription of psychotropic medications during pregnancy |  |  |  |  |
| SSRI | 1.83 (1.33, 2.56) | 1.01 (0.99, 1.03) | 1.85 (1.35, 2.61) | 2.63 (-1.51, 8.06) |
| Other antidepressants | 1.85 (1.31, 2.69) | 1.00 (0.99, 1.01) | 1.85 (1.31, 2.73) | 0.25 (-1.70, 2.80) |
| Antipsychotics | 1.84 (1.31, 2.46) | 1.00 (1.00, 1.02) | 1.85 (1.31, 2.48) | 0.87 (-0.64, 4.43) |
| Benzodiazepines | 1.85 (1.30, 2.58) | 1.00 (1.00, 1.00) | 1.85 (1.30, 2.59) | 0.12 (-0.19, 0.77) |
| Birth outcome |  |  |  |  |
| **Preterm birth** | **1.71 (1.16, 2.47)** | **1.12 (1.07, 1.17)** | **1.91 (1.26, 2.72)** | **21.83 (12.21, 37.62)** |
| Postterm birth | 1.83 (1.32, 2.53) | 1.01 (1.00, 1.02) | 1.85 (1.33, 2.55) | 1.88 (0.75, 4.42) |
| Multiple pregnancy | 1.86 (1.38, 2.55) | 0.99 (0.98, 1.00) | 1.85 (1.37, 2.52) | -1.97 (-4.50, -0.30) |
| Any birth defects | 1.97 (1.36, 3.01) | 0.92 (0.88, 0.94) | 1.81 (1.24, 2.75) | -20.17 (-51.43, -11.13) |

Bold font indicates contributory factors with % eliminated ≥5% with 95% CI not overlapping with null.

# Supplementary Table 7. Univariable decomposition analysis of excess infant mortality risk of IMD Q5 compared with Q1 for 2012-2019

|  | Natural direct effect | Natural indirect effect | Total effect | % eliminated |
| --- | --- | --- | --- | --- |
| Maternal age at childbirth |  |  |  |  |
| **<20** | **1.70 (1.19, 2.58)** | **1.09 (1.03, 1.18)** | **1.86 (1.33, 2.78)** | **18.67 (5.86, 50.55)** |
| ≥40 | 1.87 (1.33, 2.65) | 0.99 (0.96, 1.01) | 1.85 (1.32, 2.65) | -2.90 (-9.52, 2.81) |
| First live birth | 1.90 (1.37, 2.60) | 0.98 (0.97, 0.98) | 1.86 (1.34, 2.54) | -4.52 (-9.53, -3.00) |
| Prior maternal conditions |  |  |  |  |
| Anxiety | 1.84 (1.36, 2.60) | 1.00 (1.00, 1.01) | 1.85 (1.37, 2.60) | 0.62 (-0.54, 2.41) |
| **Depression** | **1.79 (1.21, 2.55)** | **1.04 (1.01, 1.08)** | **1.86 (1.27, 2.69)** | **7.92 (1.51, 21.02)** |
| Obesity | 1.83 (1.35, 2.59) | 1.01 (0.98, 1.06) | 1.85 (1.35, 2.61) | 2.52 (-6.12, 14.44) |
| Heart conditions | 1.85 (1.21, 2.58) | 1.00 (1.00, 1.00) | 1.85 (1.21, 2.58) | -0.01 (-0.09, 0.03) |
| Pregnancy factors |  |  |  |  |
| Alcohol drinking | 1.85 (1.29, 2.52) | 1.00 (1.00, 1.00) | 1.85 (1.29, 2.53) | -0.19 (-1.11, 0.59) |
| **Smoking** | **1.73 (1.29, 2.58)** | **1.07 (1.01, 1.14)** | **1.86 (1.37, 2.76)** | **14.48 (1.35, 29.05)** |
| Urinary tract infection | 1.84 (1.42, 2.71) | 1.00 (0.99, 1.02) | 1.85 (1.42, 2.74) | 0.68 (-2.44, 4.58) |
| Fever | 1.85 (1.31, 2.82) | 1.00 (1.00, 1.00) | 1.85 (1.31, 2.81) | -0.18 (-0.51, -0.06) |
| Hyperemesis gravidarum | 1.85 (1.32, 2.62) | 1.00 (0.99, 1.01) | 1.85 (1.33, 2.62) | -0.27 (-1.94, 1.82) |
| Anaemia | 1.86 (1.35, 2.71) | 1.00 (0.99, 1.00) | 1.85 (1.35, 2.70) | -0.76 (-2.01, 0.07) |
| Hypertension | 1.85 (1.36, 2.66) | 1.00 (1.00, 1.00) | 1.85 (1.36, 2.66) | -0.07 (-0.70, 0.78) |
| Pre-eclampsia | 1.85 (1.34, 2.74) | 1.00 (0.99, 1.00) | 1.85 (1.33, 2.74) | -0.35 (-1.64, 0.25) |
| Gestational diabetes | 1.85 (1.27, 2.58) | 1.00 (1.00, 1.00) | 1.85 (1.27, 2.58) | -0.06 (-0.98, 0.78) |
| Prescription of psychotropic medications during pregnancy |  |  |  |  |
| SSRI | 1.83 (1.29, 2.54) | 1.01 (0.99, 1.03) | 1.85 (1.32, 2.55) | 2.62 (-1.06, 7.96) |
| Other antidepressants | 1.85 (1.26, 2.67) | 1.00 (0.99, 1.01) | 1.85 (1.26, 2.67) | 0.28 (-1.77, 2.76) |
| Antipsychotics | 1.84 (1.32, 2.64) | 1.00 (1.00, 1.01) | 1.85 (1.33, 2.64) | 0.90 (-0.95, 3.51) |
| Benzodiazepines | 1.85 (1.29, 2.68) | 1.00 (1.00, 1.00) | 1.85 (1.29, 2.68) | 0.16 (-0.20, 0.86) |
| Birth outcome |  |  |  |  |
| **Preterm birth** | **1.71 (1.22, 2.26)** | **1.08 (1.07, 1.17)** | **1.84 (1.35, 2.59)** | **15.96 (13.04, 37.70)** |
| Postterm birth | 1.83 (1.33, 2.76) | 1.01 (1.00, 1.01) | 1.85 (1.35, 2.78) | 1.88 (0.63, 4.21) |
| Multiple pregnancy | 1.86 (1.27, 2.80) | 0.99 (0.98, 1.00) | 1.85 (1.26, 2.77) | -2.00 (-5.63, -0.09) |
| Any birth defects | 1.97 (1.43, 2.78) | 0.92 (0.88, 0.94) | 1.81 (1.32, 2.49) | -20.40 (-40.69, -12.36) |

Bold font indicates contributory factors with % eliminated ≥5% with 95% CI not overlapping with null.

# Supplementary Table 8. Univariable decomposition analysis of excess postneonatal mortality risk of IMD Q5 compared with Q1

|  | Natural direct effect | Natural indirect effect | Total effect | % eliminated |
| --- | --- | --- | --- | --- |
| Maternal age at childbirth |  |  |  |  |
| **<20** | **2.44 (1.68, 3.57)** | **1.07 (1.02, 1.13)** | **2.59 (1.78, 3.85)** | **9.95 (2.53, 21.84)** |
| ≥40 | 2.60 (2.00, 3.60) | 1.00 (0.97, 1.01) | 2.59 (2.00, 3.63) | -0.77 (-4.88, 2.26) |
| First live birth | 2.65 (1.78, 3.66) | 0.98 (0.97, 0.99) | 2.59 (1.75, 3.59) | -3.68 (-5.09, -2.30) |
| Prior maternal conditions |  |  |  |  |
| Anxiety | 2.58 (1.94, 3.56) | 1.00 (1.00, 1.01) | 2.59 (1.95, 3.56) | 0.37 (-0.34, 1.57) |
| **Depression** | **2.48 (1.80, 3.40)** | **1.05 (1.02, 1.09)** | **2.61 (1.88, 3.61)** | **7.93 (3.07, 13.70)** |
| Obesity | 2.58 (1.89, 3.68) | 1.00 (0.97, 1.05) | 2.59 (1.88, 3.72) | 0.61 (-5.53, 7.53) |
| Heart conditions | 2.59 (1.74, 3.55) | 1.00 (1.00, 1.00) | 2.59 (1.74, 3.55) | 0.00 (-0.04, 0.03) |
| Pregnancy factors |  |  |  |  |
| Alcohol drinking | 2.59 (1.82, 4.01) | 1.00 (1.00, 1.00) | 2.59 (1.82, 4.01) | 0.03 (-0.62, 0.52) |
| **Smoking** | **2.34 (1.67, 3.46)** | **1.12 (1.05, 1.20)** | **2.61 (1.86, 3.84)** | **16.82 (7.78, 29.56)** |
| Urinary tract infection | 2.58 (1.89, 3.74) | 1.00 (0.99, 1.02) | 2.59 (1.90, 3.70) | 0.49 (-1.93, 3.08) |
| Fever | 2.59 (1.90, 3.72) | 1.00 (1.00, 1.00) | 2.59 (1.91, 3.72) | 0.01 (-0.14, 0.26) |
| Hyperemesis gravidarum | 2.59 (1.88, 3.69) | 1.00 (0.99, 1.01) | 2.59 (1.89, 3.72) | 0.14 (-0.96, 1.33) |
| Anaemia | 2.59 (1.90, 3.79) | 1.00 (0.99, 1.01) | 2.59 (1.90, 3.77) | -0.17 (-0.95, 1.04) |
| Hypertension | 2.59 (1.94, 3.73) | 1.00 (1.00, 1.01) | 2.59 (1.94, 3.74) | 0.17 (-0.32, 0.79) |
| Pre-eclampsia | 2.59 (1.82, 3.54) | 1.00 (0.99, 1.00) | 2.59 (1.82, 3.53) | -0.27 (-1.14, 0.08) |
| Gestational diabetes | 2.59 (1.80, 3.77) | 1.00 (1.00, 1.01) | 2.59 (1.80, 3.78) | 0.16 (-0.30, 0.92) |
| Prescription of psychotropic medications during pregnancy |  |  |  |  |
| SSRI | 2.53 (1.87, 3.70) | 1.02 (1.01, 1.04) | 2.59 (1.90, 3.81) | 2.69 (-0.93, 6.98) |
| Other antidepressants | 2.58 (1.82, 3.68) | 1.00 (1.00, 1.02) | 2.59 (1.84, 3.71) | 0.67 (-0.83, 2.27) |
| Antipsychotics | 2.58 (1.71, 3.55) | 1.00 (1.00, 1.01) | 2.59 (1.70, 3.55) | 0.38 (-0.67, 2.10) |
| Benzodiazepines | 2.59 (1.86, 3.66) | 1.00 (1.00, 1.00) | 2.59 (1.86, 3.66) | 0.02 (-0.22, 0.30) |
| Birth outcome |  |  |  |  |
| **Preterm birth** | **2.42 (1.61, 3.47)** | **1.09 (1.05, 1.12)** | **2.63 (1.79, 3.72)** | **12.82 (8.39, 19.38)** |
| Postterm birth | 2.56 (1.90, 3.45) | 1.01 (1.01, 1.01) | 2.59 (1.92, 3.47) | 1.64 (0.85, 2.45) |
| Multiple pregnancy | 2.60 (1.85, 3.63) | 0.99 (0.99, 1.00) | 2.59 (1.84, 3.62) | -0.84 (-2.47, 0.03) |
| Any birth defects | 2.76 (1.96, 3.81) | 0.91 (0.88, 0.94) | 2.52 (1.83, 3.46) | -15.43 (-24.24, -10.26) |

Bold font indicates contributory factors with % eliminated ≥5% with 95% CI not overlapping with null.

# Supplementary Table 9. Univariable decomposition analysis of excess infant mortality risk of IMD Q5 compared with Q1 including only one child from each mother

|  | Natural direct effect | Natural indirect effect | Total effect | % eliminated |
| --- | --- | --- | --- | --- |
| Maternal age at childbirth |  |  |  |  |
| **<20** | **2.31 (1.54, 3.94)** | **1.09 (1.02, 1.17)** | **2.51 (1.71, 4.08)** | **13.20 (2.89, 29.17)** |
| ≥40 | 2.52 (1.72, 3.98) | 0.99 (0.97, 1.01) | 2.50 (1.69, 3.90) | -1.27 (-5.79, 2.69) |
| First live birth | 2.32 (1.62, 3.41) | 0.98 (0.97, 0.99) | 2.27 (1.61, 3.34) | -3.79 (-5.88, -1.91) |
| Prior maternal conditions |  |  |  |  |
| Anxiety | 2.50 (1.69, 3.66) | 1.00 (1.00, 1.00) | 2.50 (1.69, 3.67) | 0.21 (-0.30, 0.85) |
| **Depression** | **2.43 (1.63, 3.87)** | **1.04 (1.01, 1.07)** | **2.52 (1.70, 3.97)** | **5.93 (1.40, 12.95)** |
| Obesity | 2.50 (1.67, 3.53) | 1.00 (0.97, 1.05) | 2.50 (1.68, 3.54) | 0.37 (-6.35, 8.28) |
| Heart conditions | 2.50 (1.78, 3.73) | 1.00 (1.00, 1.00) | 2.50 (1.78, 3.73) | 0.01 (-0.04, 0.04) |
| Pregnancy factors |  |  |  |  |
| Alcohol drinking | 2.50 (1.58, 3.60) | 1.00 (1.00, 1.00) | 2.50 (1.59, 3.60) | 0.12 (-0.42, 0.76) |
| **Smoking** | **2.31 (1.68, 3.34)** | **1.09 (1.03, 1.17)** | **2.52 (1.83, 3.70)** | **13.87 (5.17, 25.54)** |
| Urinary tract infection | 2.51 (1.73, 3.50) | 1.00 (0.99, 1.01) | 2.50 (1.75, 3.51) | -0.25 (-2.54, 1.78) |
| Fever | 2.50 (1.76, 3.69) | 1.00 (1.00, 1.00) | 2.50 (1.76, 3.70) | 0.03 (-0.17, 0.43) |
| Hyperemesis gravidarum | 2.51 (1.65, 3.52) | 1.00 (0.99, 1.01) | 2.50 (1.64, 3.52) | -0.09 (-1.45, 1.20) |
| Anaemia | 2.51 (1.86, 3.93) | 1.00 (0.99, 1.00) | 2.50 (1.85, 3.92) | -0.31 (-1.10, 0.74) |
| Hypertension | 2.50 (1.75, 3.58) | 1.00 (1.00, 1.01) | 2.51 (1.75, 3.60) | 0.38 (-0.34, 1.20) |
| Pre-eclampsia | 2.51 (1.80, 3.65) | 1.00 (1.00, 1.00) | 2.51 (1.80, 3.65) | -0.17 (-0.70, 0.15) |
| Gestational diabetes | 2.50 (1.76, 3.41) | 1.00 (1.00, 1.01) | 2.50 (1.77, 3.42) | 0.31 (-0.36, 1.32) |
| Prescription of psychotropic medications during pregnancy |  |  |  |  |
| SSRI | 2.45 (1.70, 3.64) | 1.02 (1.01, 1.04) | 2.51 (1.72, 3.75) | 3.73 (-0.02, 8.80) |
| Other antidepressants | 2.49 (1.70, 3.67) | 1.00 (1.00, 1.01) | 2.50 (1.69, 3.69) | 0.66 (-0.85, 2.41) |
| Antipsychotics | 2.50 (1.75, 3.62) | 1.00 (1.00, 1.01) | 2.50 (1.77, 3.62) | 0.57 (-0.59, 2.42) |
| Benzodiazepines | 2.50 (1.77, 3.54) | 1.00 (1.00, 1.00) | 2.51 (1.77, 3.54) | 0.16 (-0.12, 0.76) |
| Birth outcome |  |  |  |  |
| **Preterm birth** | **2.31 (1.53, 3.41)** | **1.11 (1.06, 1.14)** | **2.56 (1.68, 3.70)** | **15.91 (9.08, 22.96)** |
| Postterm birth | 2.48 (1.75, 3.69) | 1.01 (1.00, 1.01) | 2.50 (1.77, 3.72) | 1.33 (0.57, 2.46) |
| Multiple pregnancy | 2.50 (1.69, 3.58) | 1.00 (1.00, 1.00) | 2.50 (1.70, 3.58) | 0.03 (-0.58, 0.49) |
| Any birth defects | 2.62 (1.88, 3.85) | 0.94 (0.91, 0.97) | 2.48 (1.77, 3.69) | -9.98 (-18.95, -5.74) |

Bold font indicates contributory factors with % eliminated ≥5% with 95% CI not overlapping with null.

# Supplementary Table 10. Multivariable decomposition analysis of excess infant mortality risk of IMD Q5 compared with Q1 selecting only one child from each mother

|  | **Natural direct effect** | **Natural indirect effect** | **Total effect** | **% eliminated** |
| --- | --- | --- | --- | --- |
| All contributory factors | 1.99 (1.41, 2.87) | 1.27 (1.19, 1.40) | 2.53 (1.76, 3.74) | 35.41 (26.04, 57.96) |
| Depression | 2.43 (1.66, 3.49) | 1.04 (1.01, 1.07) | 2.52 (1.72, 3.63) | 5.77 (1.46, 12.71) |
| Behaviour-related factors | 2.19 (1.51, 3.42) | 1.16 (1.06, 1.35) | 2.54 (1.75, 4.49) | 22.44 (8.73, 47.04) |
| Maternal age < 20 | 2.33 (1.53, 3.73) | 1.08 (1.02, 1.16) | 2.52 (1.69, 3.95) | 12.61 (3.27, 27.77) |
| Smoking during pregnancy | 2.35 (1.51, 3.43) | 1.08 (1.01, 1.30) | 2.54 (1.68, 4.20) | 11.97 (2.04, 35.95) |
| Preterm birth | 2.32 (1.61, 3.50) | 1.09 (1.06, 1.14) | 2.53 (1.80, 3.85) | 13.73 (9.20, 24.79) |
